# 549 Diabetic Foot Burns: A Single-Institution Retrospective Study Assessing Operative and Non-operative Outcomes

**DOI:** 10.1093/jbcr/irae036.183

**Published:** 2024-04-17

**Authors:** Brigette Cannata, Nicolas Malkoff, Deborah Choe, Artur Manasyan, Sarah Wang, Katherine Choi, Justin Gillenwater

**Affiliations:** Keck School of Medicine of USC, Staten Island, NY; Keck School of Medicine of USC, Carlsbad, CA; Keck School of Medicine of USC, Los Angeles, CA; Keck School of Medicine, University of Southern California, Los Angeles, CA; Keck Medicine of USC, Los Angeles, CA; Keck School of Medicine of USC, Staten Island, NY; Keck School of Medicine of USC, Carlsbad, CA; Keck School of Medicine of USC, Los Angeles, CA; Keck School of Medicine, University of Southern California, Los Angeles, CA; Keck Medicine of USC, Los Angeles, CA; Keck School of Medicine of USC, Staten Island, NY; Keck School of Medicine of USC, Carlsbad, CA; Keck School of Medicine of USC, Los Angeles, CA; Keck School of Medicine, University of Southern California, Los Angeles, CA; Keck Medicine of USC, Los Angeles, CA; Keck School of Medicine of USC, Staten Island, NY; Keck School of Medicine of USC, Carlsbad, CA; Keck School of Medicine of USC, Los Angeles, CA; Keck School of Medicine, University of Southern California, Los Angeles, CA; Keck Medicine of USC, Los Angeles, CA; Keck School of Medicine of USC, Staten Island, NY; Keck School of Medicine of USC, Carlsbad, CA; Keck School of Medicine of USC, Los Angeles, CA; Keck School of Medicine, University of Southern California, Los Angeles, CA; Keck Medicine of USC, Los Angeles, CA; Keck School of Medicine of USC, Staten Island, NY; Keck School of Medicine of USC, Carlsbad, CA; Keck School of Medicine of USC, Los Angeles, CA; Keck School of Medicine, University of Southern California, Los Angeles, CA; Keck Medicine of USC, Los Angeles, CA; Keck School of Medicine of USC, Staten Island, NY; Keck School of Medicine of USC, Carlsbad, CA; Keck School of Medicine of USC, Los Angeles, CA; Keck School of Medicine, University of Southern California, Los Angeles, CA; Keck Medicine of USC, Los Angeles, CA

## Abstract

**Introduction:**

Foot burns in patients with diabetes mellitus (DM) tend to be more severe with delayed presentation due to comorbid peripheral neuropathy (PN) and peripheral vascular disease (PVD). Impaired wound healing in diabetes further complicates management. Currently, there is no consensus on best management of diabetic foot burns. Our regional burn center has adopted a treatment algorithm that prioritizes non-operative local wound care instead of early excision and grafting. Here we characterize the diabetic foot burn patients treated at our institution to compare the outcomes of patients treated operatively and non-operatively.

**Methods:**

A retrospective review of all patients with DM admitted to a large urban burn center between 1/1/2015 and 6/30/2023 was conducted. Adult (age >18) patients with burns involving the foot were included for analysis. Patients with inaccessible charts and patients with burns greater than 20% total body surface area (TBSA) were excluded. In addition to descriptive statistics, linear and multinomial regression analyses were performed.

**Results:**

A total of 3,035 DM patients were identified, of which 197 (6.5%) were adults with burns involving the foot. After additional screening, 172 patients were included for analysis. The mean patient age was 56.3 years (standard deviation 11.6), and 134 (77.9%) were male. Admission hemoglobin A1C was >7% in 66.9% (n = 115) of patients. Common comorbidities included PN (44.2%), PVD (8.1%), and obesity (37.2%). The majority of burns were partial thickness (72.1%, n = 124) and affected 2.5% TBSA or less (75.6%, n = 130). The plantar foot was involved in 50% (n = 86) of cases.

Only 12.8% (n = 22) of patients were managed operatively, including debridement (9.9%, n = 17), grafting (5.8%, n = 10), and amputation (4.1%, n = 7). While 5 patients (2.9%) were primarily amputated, 2 patients (9.1%) were secondarily amputated, defined as amputation after initial debridement and grafting. Surgical management was significantly associated with increased days to wound closure (standardized coefficient = 0.251, p = 0.002) and increased length of stay (standardized coefficient = 0.576, p < 0.001). Surgical management (OR 9.3, p = 0.02, CI = 1.34 - 64.39) and peripheral neuropathy (OR 6.9, p = 0.03, CI = 1.24 - 38.68) were independent predictors of wheelchair-dependency at discharge compared to normal ambulatory status.

**Conclusions:**

Surgical management of foot burns in diabetic patients is associated with greater morbidity, including longer wound closure times, longer hospitalization, and decreased functional status at discharge. These findings suggest that local wound care of diabetic foot burns may yield better clinical outcomes than operative management.

**Applicability of Research to Practice:**

This study can be considered when recommending treatment options for diabetic foot burn patients.

